# Signaling impacts of GMO labeling on fruit and vegetable demand

**DOI:** 10.1371/journal.pone.0223910

**Published:** 2019-10-30

**Authors:** D. Adeline Yeh, Miguel I. Gómez, Harry M. Kaiser

**Affiliations:** Dyson School of Applied Economics and Management, Cornell University, Ithaca, New York, United States of America; University of Florida, UNITED STATES

## Abstract

Food labels may have both informational and signaling influences on consumer demand. We conduct a choice experiment with over 1,300 subjects to examine the signaling effect of the food product labels on consumer demand for other competing products in the market. Specifically, we focus on the genetically modified (GM) text labeling for fresh produce (strawberries, apples, and potatoes) in the United States. Contrary to some previous studies, our results indicate that the absence-claim label (Not-GM) does not have a negative impact on the demand for related conventional products. Instead, we find that consumer demand for unlabeled products is significantly enhanced with the introduction of presence-claimed GM labels. Our results contribute to the ongoing discussion of the enactment of mandatory labeling for GM foods by the federal U.S. government. Our results suggest that, in the case of direct text disclosure labels, consumers may no longer differentiate between unlabeled products and Not-GM-labeled products after the mandatory GM labeling law is in effect.

## Introduction

The agricultural and food sector has experienced astonishing technological progress over the past century, and consumers have been one of the main beneficiaries of this with an abundance of food supply at steadily decreasing real prices. At the same time, consumers have become increasingly concerned about how food is produced, processed and distributed. Production processes such as the use of genetically modified (GM) organisms (GMOs) have many critics and have prompted some consumer groups to advocate and successfully pass legislation for mandatory labels on their use in food products [1,2].

Labeling a product based on credence attributes allows producers to communicate effectively with consumers. Ideally, the informational role of food labeling should enhance consumer welfare by bridging the knowledge gap between producers and consumers, as well as by expanding the set of available options to consumers [3]. Yet, some researchers have proposed that food labels may not only have an informational role but also a signaling role, which can cast other products in a negative light. For example, the “dolphin-safe” label implicitly suggests that unlabeled conventional seafood products are produced with the harm to wildlife [4].

In the case of GM foods, consumers have long experienced choices between unlabeled conventional products and the competing “absence-claim labeled” products such as The Non-GMO Project in the marketplace. Although the industry has criticized the potential negative impacts brought by absence-claim labels to their unlabeled counterparts [5], possible signaling impacts affecting the demand for unlabeled products are not well-understood in the economic literature, particularly for fresh fruits and vegetables. Furthermore, given that the federal law of mandatory labeling for bioengineered food will soon be in effect, it is expected that the presence-claim labels of GM ingredients will be introduced to the U.S. consumers. The inclusion of such label could also create signaling impacts on the demand for other food products available in the market, which is the topic of the research summarized in this article.

To gauge potential consumer response, we conduct a behavioral analysis using a national survey of 1,306 consumers to examine the signaling effect of direct text labels on consumer choices. In the study, we ask subjects to indicate whether they would or would not purchase fresh produce products (strawberries, apples, and potatoes) based on current market prices under three different types of direct text labeling: (1) unlabeled (control group), (2) labeled with “Genetically Modified”, and (3) labeled with “Not Genetically Modified”. Through varying the ordering which participants make their purchasing choices for each label, we find that the presence-claim, GM, label induces positive signaling impact that enhances the demands for the conventional unlabeled products on the market. Surprisingly, we do not find the reverse signaling impact from the absence-claim, Not-GM, label. In other words, the demand for conventional unlabeled products is not negatively impacted by the Not-GM labels. The new federal law allows manufacturers to use several other alternative options to disclose the information on package labels including symbols, electronic links, and QR codes, but our study focuses only on direct text disclosure. Hence, our study sheds light on part of, but not the complete, set of provisions captured by the new National Bioengineered Food Disclosure Standard (NBFDS).

These results provide credence to the notion that food labels may have a signaling role to consumers. In some cases, product labels can serve as not only an identifier of the product attributes but also significantly affect the demand for other competing products on the market. The remainder of the paper is organized as follows. In the next section, we review previous studies on GM food and food labeling. This is followed by the section on the experimental design and econometric model used in the current analysis. Next, we report the results and discuss the policy implications. The paper concludes with a summary of our findings.

## Background and literature

### Genetically modified food

The introduction of GM crops in the U.S. started in the mid-1990s. GM crops generally provide production benefits to growers, such as higher pest or disease tolerance crops. Although there is no scientific evidence of GM food being harmful to human health [6], there exists a perception gap between producers and consumers on the acceptance and safety of the technology [7]. In the past decade, a vast body of literature has addressed consumer attitudes toward GM foods. While most studies elicit consumers’ willingness-to-pay (WTP) and find that consumers’ WTP for GM foods is significantly lower than for their non-GM counterparts [8–13].

Consumer aversion towards GM food has been the impetus for mandatory GM labeling proposals and laws at the state and federal levels. On December 20, 2018, the United States Department of Agriculture (USDA) announced NBFDS with the implementation date of the Standard on January 1, 2020 [14]. While a recent study suggests that WTP are different in terms of which type of disclosure is displayed [15], NBFDS allows food marketers several alternative ways to disclose the use of GMOs in food products, including the use of text or symbol directly on the food package or a QR code that consumers can use to look up the relevant information online.

In the debates over mandatory labeling, advocates focus on consumers’ “right to know” and that companies should disclose whether the product contains any GM ingredients [2,16]. However, opponents of mandatory labeling argue that it will significantly increase costs to businesses and prices to consumers as well as lead to unintended consequences [2,16–18]. For instance, given that the presence of GM ingredients must be verified at all stages of production, the cost of implementing mandatory labeling are estimated to be substantial and might be passed on to consumers in higher retail prices. Among the potential negative impacts of implementing mandatory GMO labeling, opponents are particularly concerned about consumers misinterpreting the mandatory GM labels as a warning that GM food has a higher associated risk. This leads to the discussion in food labeling literature about the signaling roles which food labels may implicitly serve.

### The informational and signaling roles of food labels

In general, the characteristics of a food production process include credence attributes that consumers cannot verify by themselves. Therefore, food labels allow companies to provide additional information about the production process to consumers in an effective manner. Nonetheless, in a broader context, food labels can potentially affect the entire food marketing system rather than simply provide information to consumers [19]. The vast majority of labeling studies focus on the “informational role” of labels, which assume that food labels are identifiers for certain product attributes such as a type of production process (e.g. shade-grown coffee, free-range eggs) [20]. In this case, consumers perceive labels only as a source of information and have different preferences for the labeled attributes. The underlying assumption is that the exposure to labels does not affect consumer demand unless the labels identify the otherwise-unknown product attributes. Consumer demand for labeled products is assumed to be exogenous to the mere presence of labels [21,22].

In addition to the informational role of food labels, in recent years, some researchers have proposed that food labels can serve not just as an identifier of attributes, but also as a signal to consumers. In other words, in addition to their informational role, food labels may have a “signaling role” that affects consumer demand. For instance, several empirical studies suggest that consumers may infer their subjective beliefs from exposure to certain food labels. A cognitive bias called the “halo effect”, which means that a consumer may biasedly evaluate some unknown or unspecified characteristics of a product due to the influence of another given attribute of that product [23], is found to be induced by some food labels. For example, in the case of the organic label, some consumers perceive it as an indication of higher quality or as being healthier even though consumers themselves are “not quite sure why” [24–26]. Schuldt, Muller, and Schwarz [27] also find similar halo effects for other credence attributes; in their case, they find that consumers view products with the fair-trade label as having lower calories.

Lusk, Schroeder, and Tonsor [28] suggest that consumer demand reflects both consumer preferences and consumer’s subjective beliefs. While preferences are traditionally considered stable or fixed, subjective beliefs are more malleable and can be affected by other information on the market. Under this framework, the case of halo effect found in consumer demand for the organic labeled products can be addressed as the following. The organic label itself informs consumers about some particular attributes (the production process), which leads to consumer preference for explicit attributes. However, the organic label also indirectly induces consumer’s subjective beliefs about other unknown product characteristics (higher quality or healthier) [25]. These studies indicate that food labels may have both an informational role and a signaling role, where the informational role affects consumer preference, while the signaling role relates to consumer subjective beliefs.

The above empirical studies focus on the signaling roles of food labels that induce demand responses for the product itself. However, the impacts brought by the signaling role of food labels may further affect the subjective beliefs of other products available in the market. For example, “certified humane” implicitly suggests the unlabeled products are produced inhumanely, and the “low sodium” label may be viewed as a warning that salt should be avoided [3,29]. These signaling effects occur when consumers receive an implicit message from an explicit cue [29]. Conventional producers have raised concerns about the negative signaling impacts brought by food labels [5]. The negative signaling impacts brought by a certain label to other items in the same product category can be referred to the psychological concept of stigma. Stigma indicates a negative mark on an attribute that pervades an otherwise acceptable entity [30]. For instance, according to Kanter, Messer, and Kaiser [31], although rBST is a synthetically produced natural hormone that the FDA declares is not harmful to human health, the “rBST-free” milk label stigmatizes consumer demand for conventional unlabeled milk. While the industry has raised concerns about absence-claim labels stigmatizing conventional products, the concept of stigmatization from food labels has not yet been thoroughly studied in the economic literature, particularly in fresh produce.

### The signaling effects of GM and Not-GM labels

Signaling effects brought by GM labels can be of two types: absence-claim labels and presence-claim labels. First, absence-claim labels of GM ingredients have been in the marketplace for a long period of time. Some products are certified voluntarily under the Non-GMO Project or the organic certification, where both certifications restrict the use of GMO ingredients, while some simply include a text on the package indicating the absence of GM ingredients without formal certification [32,33]. Even though consumers have been exposed to absence-claim labels (Not-GM-labeled products) for several years, we know little about possible stigmatization effects caused by absence-claim labels. It is still unclear whether the absence-claim labels for GM products stigmatize conventional products as is the case of rBST-free milk label that was tested in Kanter, Messer, and Kaiser [31].

Moreover, once the NBFDS is enacted, the presence-claim label of GMOs will be introduced to the market. A recent study suggests the heterogeneous consumer characteristics between consumers who sought to avoid GM presence label with consumers who sought to choose a GM absence label [34]. The study further implies that the GM presence labels established by the NBFDS along with the existing GM absence labels may nudge consumer choices. From the viewpoint of signaling impacts on demand, potential signaling impacts from the newly introduced presence-claim GM labels may spill over to the demand for other products available in the market. In other words, as the reverse concept of stigmatization brought by the absence-claim label, the presence-claim GM label can potentially enhance the demand for conventional unlabeled products. These possible signaling effects of GM labels have not been thoroughly examined in the literature. Also, the few studies conducted in related settings have found somewhat conflicting results. For example, Lusk and Rozan [35] suggest that consumers are less willing to consume GM food if they believe mandatory labeling law exists, while Costanigro and Lusk [20] find little evidence of signaling effects from GM labels on consumer’s subjective beliefs and Kolodinsky and Lusk [36] find that the short-term mandatory GM labeling in Vermont led to less opposition to GM food. Besides, according to Liaukonyte et al. [37], consumers have asymmetric sensitivity in WTP between “contains” and “free of” labels in general. Thus, even if signaling effects exist for both absence- and presence-claim labels, the magnitude of impacts could be substantially different.

Therefore, in the case of GM food, this study sheds light on the economics of consumer demand by testing the signaling role of the absence-claim and presence-claim food labels, focusing on fresh produce. We quantitively evaluate the potential signaling effects induced by Not-GM and GM direct text labels through the viewpoint of decreasing and enhancing the demand for other products on the market. Also, this research contributes to the literature through assessing impacts brought by the signaling roles of food labels on other products in the market instead of to the labeled product itself. Finally, given that the mandatory labeling law will soon be in effect, our results provide policy-relevant estimates on the potential demand shifts brought by the GM label to other competing product options available in the market.

## Methods

### Experimental design

The main contribution of this study is to measure the signaling effects brought by absence-claim and presence-claim GM labels in fresh produce. The experimental design focuses specifically on direct text disclosure, which is one of the several options allowed by NBFDS to disclose GM ingredients on the food package. In order to evaluate the signaling effects of GM related labels, the key experimental design is the sequential presentation of products. The signaling effects are evaluated under the changes in demands between different orders of the choice sets. The details and rationale of the experimental design are as follow.

The consumer choice experiment is designed as an online survey and conducted via Qualtrics. We ask participants to indicate whether they are willing to purchase a product, given a generic product picture and a market price. The product is presented either unlabeled (control group) or with one of two text labels: (1) “Genetically Modified” (GM), and (2) “Not Genetically Modified” (Not-GM). For simplicity, hereafter we use the term “type of labeling” to refer either the control group (unlabeled) or the two labels (GM and Not-GM). We select three fresh produce products which have USDA-approved GM varieties: strawberries, apples, and potatoes. We include only fresh produce in the survey, considering the lack of relevant research in this area and that processed food with the GM label is ambiguous about the amount of GM ingredients contained.

The experimental design is similar to Kanter, Messer, and Kaiser [31] by varying the order of choices in each treatment. In this study, participants are randomly assigned to one of six treatments. Each treatment varies according to the sequence in which types of labeling are presented in the set of choices. Participants in Treatment 1 receive the purchasing choice questions for all the GM-labeled products first, followed by the unlabeled conventional products. In Treatment 2, participants are shown the GM-labeled products first, followed by the Not-GM-labeled products instead. And so on for the other treatments. Table 1 lists the complete orderings of labels for all six treatments. The only difference between treatments is the order in which the three types of labeling are presented while the presentation of products (strawberry, apple, and potato) within the same type of labeling group is randomly ordered. Participants in each treatment answer a total of six label-product choice questions.

**Table 1:** The ordering of labels for each treatment.

Treatment #	Label presented		Number of participants (random assignment)
	First	Second	
1	GM	Unlabeled	208
2	GM	Not-GM	214
3	Unlabeled	GM	223
4	Unlabeled	Not-GM	215
5	Not-GM	GM	221
6	Not-GM	Unlabeled	225

**Fig 1:**
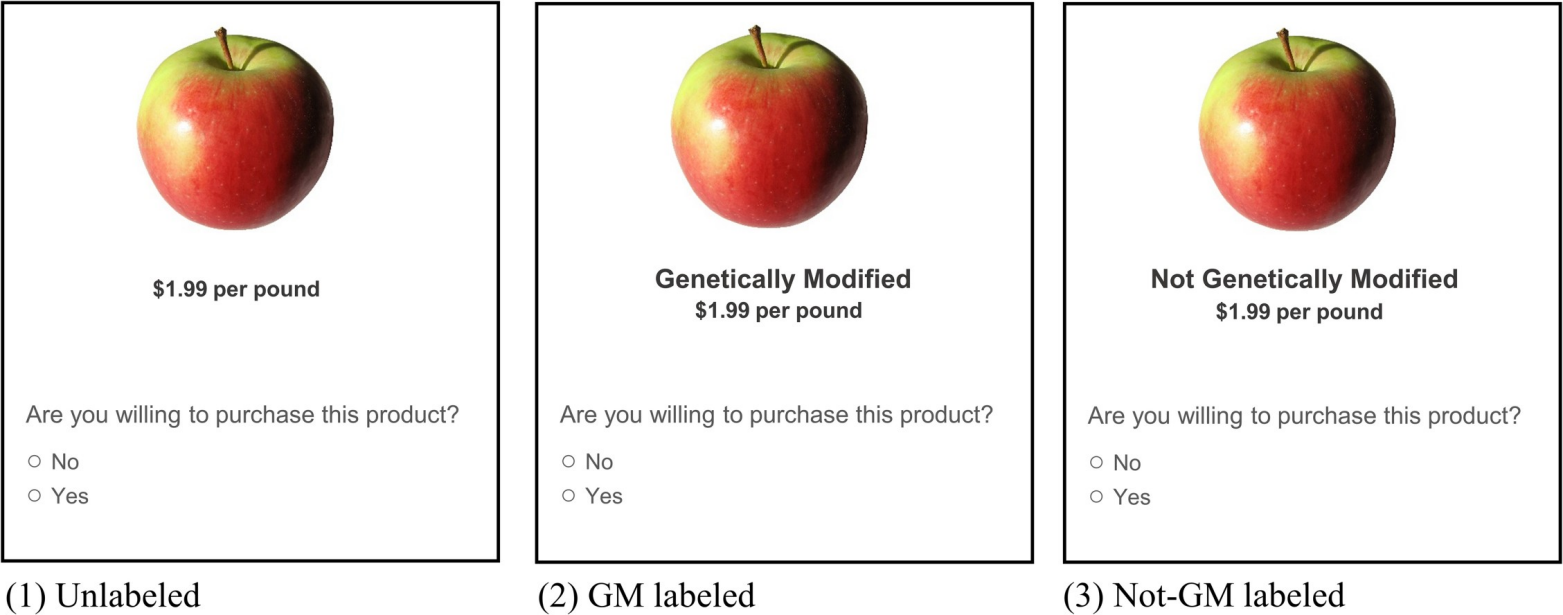
Example questions in the survey. (A) Each question is shown on a separated page of the survey. (B) The product figure is similar but not identical to the original image and is therefore for illustrative purposes only. Photo credit: “FreeImages.com/photographer/bury-osiol-59143”.

The choice of each labeled product is presented on a separate page, and participants cannot change their previous responses once they move to the next page. Sample questions in the survey are shown in Fig 1 for the case of apples. For all three types of labeling, the listed market prices are the same and based on the average retail prices at the time the survey was conducted. Participants proceed to the demographic section of the survey after they complete the choice questions. The demographic section includes questions of the socio-demographic characteristics and other behavioral questions regarding food purchase, attitude toward related issues, etc.

Given that the objective of this study is to understand how consumer demand of a given type of labeling can be affected by the signals induced by other types of labeling, we need to first define the baseline demand of the given type of labeling as the comparison basis. We use the average estimated willingness-to-buy (WTB) from the treatments which receive that type of labeling first. We refer this demand baseline as the “initial demand” for each label-product. The initial demands for the GM-labeled products are estimated from the choices made by participants in Treatments 1–2. The initial demands for unlabeled products and Not-GM-labeled products are estimated from Treatments 3–4 and Treatments 5–6, respectively. All participants entered the survey seeing the same generic description at the beginning and were randomly assigned to one of the six treatments. The nature of the experiment design was not revealed to the participants. Thus, the first purchasing choices participants made are only affected by the type of labeling displayed but not affected by any other different information across treatments.

To evaluate whether one type of labeling positively or negatively affects the demand of other types of labeling, the experiment is designed for between-treatments demand comparison, which is similar to Kanter, Messer, and Kaiser [31]. We estimate whether the choices made for the products with the given type of labeling in other treatments, where participants receive the type of labeling in the second order, significantly increase or decrease from the initial demand of the given type of labeling. If so, this indicates that consumer demand for the given type of labeling is affected by the presentation order of the label. If one is interested in eliciting the demand for a label, the experiment can simply present each treatment with only one of the three labels and compare the WTB between treatments. In this study, varying the ordering of presented labels allows us to evaluate whether the label presented at first induces signaling impacts that affect consumer’s preferences for other labeled or unlabeled products in the second order. Furthermore, by revealing labels sequentially, this experimental setting mimics an expansion of product options available on the market. Thus, the experimental setting allows us to answer the policy-relevant question of how the introduction of products with text disclosure of GMOs might impact unlabeled or Not-GM-labeled products available on the market.

The prices are purposely kept at the same level for all three types of labeling in the experimental design. If we include price variations across GM, Not-GM and unlabeled products, we cannot clearly distinguish whether the demand changes are caused by the price effects or the induced signaling effects. Also, the bioengineered fresh produces currently on the market have other positive product attributes, such as the non-browning trait of Arctic apple, and do not have a clear indication of price discounts or premiums. Thus, we do not possess enough information to determine the price difference for GM labeled products. One could argue that participants may expect a higher price of Not-GM goods from their experience in the real marketplace and may thereby intend to buy the Not-GM products due to the same price design. However, given that the main analysis in this study is a between-treatments comparison, with random assignment to each treatment, even if the bias exists, the underlying assumption is that the bias exists across all treatments and will be canceled out by the between-treatments comparison. Another concern one may have is the possible hypothetical bias that is often raised in discrete choice analyses. Hypothetical bias arises in stated preference valuation research when participants report a higher willingness to pay or make decisions that they normally would not make in real life [38–40]. Nonetheless, given that we are only interested in the differences between treatments and not in eliciting the actual WTB, the maintained assumption here is that there is no interaction between hypothetical bias and the treatment assignment.

### Data and econometric model

A total of 1,306 effective responses were obtained by an online survey software provider, Qualtrics. We only recruited participants over 21 years old and the survey included screening questions to exclude non-attentive responses. Table 1 lists the number of respondents by treatment. Given that participants were randomly assigned to one of the six treatments, there are slightly different total counts of respondents in each treatment, ranging from 208 to 225 participants per treatment.

Table 2 summarizes the behavioral variables, socio-demographic characteristics of the sample, and provides a comparison with the 2010 U.S. Census for face validity purposes. Given that we restricted the survey to respondents aged 21 and higher, the median age of the sample is age 52, which is higher than the national median age of overall U.S. population in the 2010 Census. Nevertheless, the sample has adequate representation across all age categories. The shares of each age group in our sample is very close to the 2010 Census, except for the 60–69 age group. Regarding gender, 74% of respondents are female in our sample, which is reflective of females being the primary food shopper in U.S. households but slightly higher than the national share of the female population in the 2010 Census. Furthermore, the sample is slightly biased toward higher educated respondents relative to the Census, because the survey was implemented online. The sample is representative geographically of the U.S. population. Regarding the behavioral variables, in general, participants do concern about different food-related issues and spend on average 28% of their food purchase on organic food. We perform Tukey’s tests on group means and find no significant difference between the six treatments on any socio-demographic or behavioral variables.

**Table 2:** Summary statistics for demographic and behavioral variables with comparison to the 2010 U.S. Census.

	Survey	2010 Census ^a^
Number of total respondents	1,306	--
Median Age	52 (include only age 21+)	37.2 (all U.S. population)
Split between age groups:	*Percentage to total respondents*	*Percentage to age 21+ population*
21–29	13.2%	17.3%
30–39	17.6%	18.2%
40–49	16.4%	19.7%
50–59	19.1%	19.0%
60–69	22.6%	13.2%
70 up	11.2%	12.6%
	*Percentage to total respondents*	*Percentage to total population*
Female	74.3%	52.0%
With children under 18 in the household	28.6%	29.8%
Primary food shopper	90.1%	--
Vegetarian or vegan	4.8%	--
Education	*Percentage to total respondents*	*Percentage to age 18+ population*
Less than high school	1.8%	13.7%
High school	21.8%	31.0%
College or associate degrees	64.4%	46.0%
Postgraduate	11.9%	9.3%
Geographic Regions ^b^	*Percentage to total respondents*	*Percentage to total population*
Northeast: New England	5.7%	4.7%
Northeast: Middle Atlantic	12.9%	13.2%
Midwest: East North Central	16.0%	15.0%
Midwest: West North Central	6.9%	6.6%
South: South Atlantic	20.8%	19.4%
South: East South Central	6.8%	6.0%
South: West South Central	8.5%	11.8%
West: Mountain	7.8%	7.2%
West: Pacific	14.6%	16.2%
	*Average across all respondents*	
Percentage of organic food purchase	28.09%	--
Political ideology (extremely conservative = 7)	4.36	--
Concern about animal welfare (1 to 7)	5.47	--
Concern about food safety (1 to 7)	5.94	--
Concern about pesticide residues in fresh produce (1 to 7)	5.70	--
Subjective knowledge about GM food (extremely knowledgeable = 7)	3.60	--

^a^ Source: U.S. Census Bureau; calculated by authors using American FactFinder (https://factfinder.census.gov/faces/nav/jsf/pages/index.xhtml)

^b^ The geographic regions follow divisions from the U.S. Census Bureau. The states categorized in each region can be found at https://www2.census.gov/geo/pdfs/maps-data/maps/reference/us_regdiv.pdf

In the survey, participants answered the dichotomous yes/no choice on a posted market price for each label-product. In the econometric model, the subscript *i* refers to the participant, and *L* denotes one of three types of labeling: unlabeled (*un*), GM-labeled (*GM*), and Not-GM-labeled (*NGM*). The binary purchasing decision for a product with one type of labeling (*L*) is thereby denoted as *y*_*i*.*L*_:

$$y_{i.L}=\{\begin{array}{l} 1\;\mathrm{if}\;\mathrm{participant}\;i\;\mathrm{chooses}\;\mathrm{to}\;\mathrm{purchase}\;\mathrm{the}\;\mathrm{product}\;\mathrm{with}\;\mathrm{type}\;\mathrm{of}\;\mathrm{labeling}\;L\\ 0\;\mathrm{otherwise} \end{array}$$ (1)

Under the random utility framework [41], we employ a logit model using maximum likelihood estimation. The probability of participant *i* buying the product with type of labeling *L* (i.e. *y*_*i*.*L*_ = 1) is defined as *P*_*i*,*L*_. Eq (2) specifies the logit function of *y*_*i*.*L*_, which refers to the natural log of the odds that *y*_*i*.*L*_ equals to one. The error term *ϵ*_*i*,*L*_ is assumed to be identically and independently distributed extreme-value.

$$\begin{array}{ll} \log\left(\frac{P_{i,L}}{{1-P}_{i,L}}\right) & =\alpha_{0,L}+\alpha_{1,L\in \{un,NGM\}}\cdot After\_GM+\alpha_{2,L\in \{un,GM\}}\cdot After\_NGM\\ & \;+\beta_{p,L}\cdot {products}_{p}+\sum_{k}\gamma_{k,L}{Demo}_{ik}+\epsilon_{i,L}\\ & =X_{i}\prime \delta_{L}+\epsilon_{i,L} \end{array}$$ (2)

$${\hat{P}}_{i,L}=\frac{exp{(X}_{i^{\prime }}\delta_{L})}{1+exp{(X}_{i^{\prime }}\delta_{L})}$$ (3)

Given that we are interested in the effects of labeling overall but not interested in each product per se, we group all products into one model to increase the power of estimation. We also estimated for product-specific models, and the results from each product-specific model are consistent and of similar magnitude across products (not presented here). A total of three logit models are estimated for three types of labeling. The variables of interest, *After_GM* and *After_NGM*, are dummy variables indicating whether the given type of labeling *L* is shown to the participant after the GM-labeled products or after the Not-GM-labeled products. We fix strawberry as the reference product and include dummy variables, *product*_*p*_, where *p* denotes the product set including apple and potato. The vector *Demo*_*ik*_ denotes demographic characteristic *k* for individual *i* as listed in Table 2. Eq (3) refers to the predicted probability of the buying (${\hat{P}}_{i,L}$), or predicted WTB of the given type of labeling, under the logit model. The vector of covariates (*X*_*i*_) and the corresponding coefficient vector (**δ**_*L*_) includes *After_GM*, *After_NGM*, *product*_*p*_ and *Demo*_*ik*_.

The hypotheses on the labeling induced signaling impacts can be expressed as:

$$H_{1}:\;\alpha_{1,L\in \{un,NGM\}}>0$$ (4)

$$H_{2}:\;\alpha_{2,L\in \{un,GM\}}<0$$ (5)

The two coefficients *α*_1,*L*_ and *α*_2,*L*_ capture the signaling effects which the GM or Not-GM label would induce. The first hypothesis is that, if the presence-claim label, GM, enhances the demand for the other products (unlabeled or Not-GM-labeled), then *α*_1,*L*_ should be positive and statistically significant. The second hypothesis, on the other hand, suggests that if the absence-claim label, Not-GM, decreases the demand for other products (unlabeled or GM-labeled), then *α*_2,*L*_ should be negative and statistically significant.

## Results

Table 3 reports the estimated marginal effects for the changes in WTB from the proposed econometric model. A total of three logit models are included as models 1–3 in Table 3. The three models refer to the purchasing probabilities in percentage terms (i.e. WTB) for unlabeled, Not-GM-labeled, and GM-labeled products, respectively. The marginal effects are the changes in WTB brought by increasing a unit of an explanatory variable while holding all other explanatory variables at the sample mean levels. Therefore, the marginal effects of the dummy variables, *After_GM* and *After_NGM*, measure the changes in WTB compare to initial demand when the product is presented after GM-labeled or Not-GM-labeled products. In other words, the posted marginal effects of *After_GM* and *After_NGM* refer to the signaling effects brought by the presence-claim and absence-claim labels to the demands for other products on the market. As previously mentioned, the change in marginal effects is with regard to the initial demand, which is assumed to be the average WTB when the type of labeling presented to participants first in the sequence of choices. For example, in model 1, the initial demand for unlabeled products is estimated from Treatment 3–4 (unlabeled were shown first); the signaling impact induced by GM label is estimated from Treatment 1 (presented GM products then unlabeled products); and the signaling impact induced by Not-GM label is estimated from Treatment 6 (presented Not-GM products then unlabeled products).

**Table 3:** Estimated marginal probabilities of logit models.

	(1)	(2)	(3)
Purchasing decisions for	Unlabeled products	Not-GM-labeled products	GM-labeled products
*After_GM*: Presented after GM label	0.122***	0.015	--
	(0.021)	(0.019)	--
*After_NGM*: Presented after Not-GM label	0.019	--	-0.019
	(0.022)	--	(0.023)
Product = apple	0.091***	0.092***	0.056**
	(0.022)	(0.023)	(0.026)
Product = potato	0.216***	0.164***	0.146***
	(0.021)	(0.022)	(0.026)
Female	-0.030	-0.018	-0.080***
	(0.021)	(0.020)	(0.026)
Age	-0.004***	-0.003***	-0.005***
	(0.001)	(0.001)	(0.001)
With children under 18 in the household	0.011	0.054**	0.015
	(0.022)	(0.021)	(0.026)
Primary food shopper	-0.075***	0.011	-0.076*
	(0.028)	(0.032)	(0.039)
Vegetarian or vegan	-0.002	-0.125***	0.040
	(0.046)	(0.044)	(0.048)
Percentage of Organic food purchase	0.001***	0.002***	-0.001***
	(0.000)	(0.000)	(0.000)
Political ideology (extremely conservative = 7)	0.015***	0.003	-0.023***
	(0.006)	(0.006)	(0.007)
Concern about animal welfare	0.029***	0.006	0.018**
	(0.007)	(0.007)	(0.008)
Concern about food safety	0.005	0.017*	-0.032**
	(0.010)	(0.010)	(0.013)
Concern about pesticide residues	-0.012	0.009	-0.022**
	(0.009)	(0.009)	(0.011)
Subjective knowledge for GM food (extremely knowledgeable = 7)	-0.014**	-0.001	-0.007
	(0.006)	(0.006)	(0.007)
Region Fixed Effects for 9 U.S. regions	Y	Y	Y
Education Fixed Effects	Y	Y	Y
Total number of choices	2,613	1,980	1,929
Treatments included for initial demand	3 and 4	5 and 6	1 and 2
Treatments included for signaling impacts	1 and 6	2	5

*Notes*: Reported results are estimated marginal probabilities. In the binary logit model, the marginal probability refers to the change in probability of y = 1 brought by a given explanatory variable when measured at the mean of all other explanatory variables. Numbers in parentheses are standard errors.

*** p<0.01

** p<0.05

* p<0.1. We test the models with the addition of interaction effects between locations and treatments as well as educational levels and treatments. We do not find significance in the interaction terms and other estimates are consistent.

For unlabeled products (model 1 in Table 3), results show that participants are 12.2% more likely to purchase unlabeled products when the unlabeled products are shown after the GM-labeled products. In other words, we find a signaling effect of the GM-label that boosts the demand for unlabeled products. However, when unlabeled products are presented after the Not-GM-labeled products, the demand for unlabeled products is not significantly different relative to the initial demand. Thus, there is no evidence of negative effects of the Not-GM label on the unlabeled, conventional products. Regarding Not-GM-labeled and GM-labeled products, the results suggest that the presentation order does not significantly affect WTB (models 2 and 3). These results show that signaling effects only exist in certain cases. We discuss the signaling impacts and the implications in-depth in the next section.

Regarding other socio-demographic and behavioral variables, the directions of the estimated marginal effects are generally consistent with the consumer literature. Across three labels, potato has the highest WTB, followed by apple and strawberry. In terms of age, older participants are less willing to buy the given products across all three labels. Gender only plays a role in the purchasing decision of GM-labeled products, where females are 8% less likely to purchase GM-labeled food than males. Consumers with children are 5.4% more willing to buy Not-GM-labeled products than the rest. Primary food shoppers are 7.5% less likely to buy the given unlabeled products and 7.6% less willing to purchase GM-labeled products relative to non-primary food shoppers. Consumers that purchase more organic food are slightly more likely to buy Not-GM-labeled or unlabeled products and less likely to buy GM-labeled products than the rest. Consumer attitudes of various food-related issues also contribute to their purchasing decisions. For instance, individuals concerned about food safety exhibit higher WTB for Not-GM-labeled products but lower WTB for GM-labeled products than non-concerned respondents. In addition, consumers concerned about pesticide residues are 2.2% less likely to buy GM-labeled products than the rest. Interestingly, the subjective knowledge of GM food does not significantly affect consumers’ demand for Not-GM-labeled or GM-labeled products.

## Discussion

Table 4 shows the average WTB from the raw data and the predicted WTB using estimates from Table 3 holding all other covariates at their mean levels. The predicted WTB are close to actual WTB from raw data, which means presentation order is the main driver of the changes in WTB. The average predicted initial demand for Not-GM-labeled products is the highest (78.7%), followed by unlabeled products (65.2%) and GM-labeled products (41.5%). In this section, we discuss how the predicted WTB changes as we manipulate the order of product presentation.

**Table 4:** Average willingness-to-buy (WTB) across products under the given label and presented order.

	Average from the raw data			Predicted from the Logit model ^a^		
	Unlabeled	Not-GM-labeled	GM-labeled	Unlabeled	Not-GM-labeled	GM-labeled
Initial demand (presented first)	64.6%	76.7%	41.6%	65.2%	78.7%	41.5%
Presented after GM	77.1%	79.1%	--	77.7%	80.3%	--
Presented after Not-GM	66.1%	--	41.2%	67.2%	--	39.5%

^a^ Results are calculated using estimates from the three logit models (Table 3). Other socio-demographic and behavioral covariates are held at sample means. The estimates are averaged across three products.

First, the results on testing the signaling effects induced by absence-claim and presence-claim labels are somewhat surprising. The results indicate that the absence-claim label (Not-GM) does not decrease the demand for either the unlabeled conventional products or the GM-labeled products, at least in fresh produce. The average demand for unlabeled products is 65.2% initially and 67.2% when presented after Not-GM-labeled products, where the difference between demands is statistically insignificant. For the case of GM-labeled products, the average willingness-to-buy is 41.5% initially and 39.5% when presented after Not-GM-labeled products. The change between the two estimated demands is also not statistically significant. This finding is opposite to the case of rBST-free milk label analyzed in Kanter, Messer, and Kaiser [31], where they found significant stigmatization of conventional milk as a result of the introduction of rBST-free labeled milk. This contrasting result is perhaps due to the fact that Not-GM-labeled products have been in the market for a long period of time. Thus, consumers are used to the existence of Not-GM-labeled products and might only view the Not-GM label as an identifier for the product attribute rather than signaling the characteristics of other products.

On the other hand, the GM-label has never been used in the U.S. market (except for a very short period in Vermont). The results show significant signaling effect for GM labels to unlabeled products. As shown in column 1 of Table 4, the demand for conventional unlabeled products increases from 65.2% initially to 77.7% if they are presented after the GM-labeled products. We note that the WTB for unlabeled conventional products presented after GM labels increased to about the same level as the initial demand for Not-GM-labeled products (78.7%). This result suggests that consumers may not differentiate between unlabeled products and Not-GM-labeled products after the introduction of GM labels. Again, this may be due to the fact that consumers are already very familiar with the Not-GM label as an identifier for the product attribute rather than signaling the characteristics of other products. If so, it might not be worthwhile for companies to go through formal Not-GM certifications after the implementation of the GM labeling law. In contrast, the average WTB for Not-GM-labeled products is only modestly affected after the introduction of GM labels (78.7% initially versus 80.3% after introducing the GM labels).

We find that consumer demands are consistent for both the specified GM-labeled products and the Not-GM-labeled products, regardless of the order in which the labeled products are presented. These results suggest that consumer demands for labeled products tested in this study are not affected by other product options available in the market. Thus, they may be relatively insensitive in their purchasing decisions for GM-labeled and Not-GM-labeled product. In other words, consumer preferences are not much amendable by the implicit information signaled by other products in the market.

In addition, the initial demands in Table 4 suggest an interesting asymmetry in predicted WTB between the unlabeled control and absence-claim label as well as between the unlabeled control and the presence-claim label. If we take the predicted initial demand for unlabeled products as a reference for comparison, the predicted WTB shows the asymmetric changes in initial WTB for the presence-claim label and the absence-claim label. Absence-claim label results in an increase of 13.5% in WTB (i.e. difference between 65.2% and 78.7%), while presence-claim label has a larger decrease of 23.7% in WTB (i.e. difference between 65.2% and 41.5%). The demand response of facing GM-labeled products is almost twice the size in decrease than the increase when facing Not-GM-labeled products. This reflects one of the human behavioral anomalies, namely that negative information has a greater influence on consumers than positive information has [42]. Since consumers generally view GM as a negative product characteristic, the GM label conveys negative information while Not-GM label conveys positive information to consumers. The asymmetric demand responses in the context of GM and Not-GM labels are consistent with the estimates in Liaukonyte et al. [37] and Costanigro and Lusk [20].

## Conclusion

In this study, we used an experimental approach to examine the potential signaling impacts of presence-claim and absence-claim text labels regarding GM fresh produce. The experimental results support the general findings in previous literature that food labels can be more than just an identifier of product attributes, but also a signal influencing consumer preference for alternative products available on the market. The results show that consumer demand for unlabeled products may be enhanced with the introduction of presence-claimed GM text labels, which will soon be on the market due to the enactment of mandatory labeling by the federal government. In contrast, results suggest that consumer demand for GM-labeled and Not-GM-labeled products are relatively fixed regardless of the available options on the market. Although some of the behavioral studies suggest that absence-claim labels stigmatize conventional, unlabeled products, we do not find such effects in the case of Not-GM text labels in fruit and vegetable demand. Moreover, the substantial asymmetric differences in demand between absence-claim and presence-claim labels to the demand for unlabeled products support the existence of asymmetric negativity effect raised in consumer literature in the case of GMOs.

This study contributes to the behavioral literature by providing experimental evidence of the signaling role brought by fresh produce text labels in addition to their informational role and illustrate the potential influence on the demands for other products in the market. Our results shed light on the impacts of the forthcoming mandatory GM labeling legislation by providing the potential signaling impacts after introducing a new presence-claim GM text label to the market. One major marketing implication of this result is that when GM-labeling is mandatory, non-GM products may not need the not-GM label because consumers will perceive non-labeled products as not containing GMs, which will of course be true. Hence, in the case of fresh produce, the existence of mandatory labels will mitigate the need for not-GM labels for non-GM products. This will reduce supply chain costs, in particular to growers focusing on not-GM produce, because they generally bear the costs of certifications and labels. Another marketing implication of our results is that GM fresh produce growers must ensure that the positive attributes brought by the GM technology (e.g. avoiding rapid browning in apples) offset the negative effect on demand caused by the mandatory GM label.

Several caveats should be noted which suggest the need for future research. First, the choice experiment in this study includes only fresh fruit and vegetables. Although there has been an increasing number of GM fruit and vegetables introduced to the market in recent years, the majority of fresh produce currently available in the marketplace is not bioengineered. Thus, the signaling impacts may be different if the featured products are processed food or other food products with widely recognized GM varieties adoption such as canola oil or soybean products. Secondly, the experimental design focuses specifically on text disclosure but does not take into account other disclosure options allowed by the NBFDS such as symbols or QR codes. The results of this study thus apply only to text disclosure labels, but not to other disclosure label options. Given that text disclosure is generally more noticeable to consumer and the term “bioengineered” specified in NBFDS is less recognizable to consumers relative to the term “genetically modified” used in this study, the estimated signaling effects can be viewed as the upper bounds of the true effects. Future research should assess other disclosure options allowed under the NBFDS. Finally, consumers generally perceive different types of labeling for the same product side by side in the grocery stores. However, such labels are not found in grocery stores yet and the best alternative is to use the sequential introduction of the labels in the experimental design to evaluate the signaling impacts from the ordering effects of choices [31,43]. Future research can examine the impacts of GM labels of consumer choices in actual shopping occasions when fresh produce with such labels are available in grocery stores.

## Supporting information

S1 Dataset Dataset. (CSV)

## References

[1] Pino D. Why I’m Voting Yes on Prop 37: Label Genetically Modified Foods. Huffington Post [Internet]. 2012 10 29 [cited 2018 Jan 15]; Available from: https://www.huffingtonpost.com/darya-pino/prop-37-genetically-modified-food_b_2040371.html

[2] Huffman WE, McCluskey JJ. The Economics of Labeling GM Foods. AgBioForum. 2014 12 23;17(2):156–60.

[3] Messer KD, Costanigro M, Kaiser HM. Labeling Food Processes: The Good, the Bad and the Ugly. Appl Econ Perspect Policy. 2017 9 1;39(3):407–27.

[4] Watson KW. “Dolphin Safe” Labels On Canned Tuna Are A Fraud. Forbes [Internet]. 2015 4 29 [cited 2018 Sep 4]; Available from: https://www.forbes.com/sites/realspin/2015/04/29/dolphin-safe-labels-on-canned-tuna-are-a-fraud/

[5] Vilsack T. Stop the Food Label Fear-Mongering [Internet]. US News & World Report. 2018 [cited 2018 Jul 15]. Available from: https://www.usnews.com/opinion/articles/2018-01-30/stop-the-food-label-fear-mongering

[6] National Academies of Sciences. Genetically Engineered Crops: Experiences and Prospects. Washington, DC: The National Academies Press; 2016. 28230933

[7] Lusk JL, McFadden BR, Wilson N. Do consumers care how a genetically engineered food was created or who created it? Food Policy. 2018 7 1;78:81–90.

[8] Lusk JL, Jamal M, Kurlander L, Roucan M, Taulman L. A Meta-Analysis of Genetically Modified Food Valuation Studies. J Agric Resour Econ. 2005;30(1):28–44.

[9] Bernard JC, Zhang C, Gifford K. An Experimental Investigation of Consumer Willingness to Pay for Non-GM Foods When an Organic Option Is Present. Agric Resour Econ Rev. 2006 10;35(2):374–85.

[10] Dannenberg A. The dispersion and development of consumer preferences for genetically modified food—A meta-analysis. Ecol Econ. 2009 6 15;68(8):2182–92.

[11] Frewer LJ, van der Lans IA, Fischer ARH, Reinders MJ, Menozzi D, Zhang X, et al Public perceptions of agri-food applications of genetic modification–A systematic review and meta-analysis. Trends Food Sci Technol. 2013 4 1;30(2):142–52.

[12] Fernandez-Cornejo J, Wechsler S, Livingston M, Mitchell L. Genetically Engineered Crops in the United States [Internet]. Washington, DC: Economic Research Service, United States Department of Agriculture; 2014 2 [cited 2018 Apr 14]. Report No.: ID 2503388. Available from: https://papers.ssrn.com/abstract=2503388

[13] Hess S, Lagerkvist CJ, Redekop W, Pakseresht A. Consumers’ evaluation of biotechnologically modified food products: new evidence from a meta-survey. Eur Rev Agric Econ. 2016 12 7;43(5):703–36.

[14] United States Department of Agriculture. National Bioengineered Food Disclosure Standard [Internet]. 2018 [cited 2019 Jan 11]. Available from: https://www.ams.usda.gov/rules-regulations/be

[15] McFadden BR, Lusk JL. Effects of the National Bioengineered Food Disclosure Standard: Willingness To Pay for Labels that Communicate the Presence or Absence of Genetic Modification. Appl Econ Perspect Policy. 2018 6 1;40(2):259–75.

[16] Golan E, Kuchler F, Mitchell L, Greene C, Jessup A. Economics of Food Labeling. J Consum Policy. 2001 6 1;24(2):117–84.

[17] Oh J, Ezezika OC. To label or not to label: balancing the risks, benefits and costs of mandatory labelling of GM food in Africa. Agric Food Secur. 2014 4 23;3:8.

[18] Hemphill TA, Syagnik B. Genetically Modified Organisms and the U.S. Retail Food Labeling Controversy: Consumer Perceptions, Regulation, and Public Policy. Bus Soc Rev. 2015 9 2;120(3):435–64.

[19] Caswell JA, Padberg DI. Toward a More Comprehensive Theory of Food Labels. Am J Agric Econ. 1992 5;74(2):460.

[20] Costanigro M, Lusk JL. The signaling effect of mandatory labels on genetically engineered food. Food Policy. 2014 12 1;49:259–67.

[21] Bansal S, Chakravarty S, Ramaswami B. The informational and signaling impacts of labels: experimental evidence from India on GM foods. Environ Dev Econ. 2013 12;18(6):701–22.

[22] Liaukonyte J, Streletskaya NA, Kaiser HM. Noisy Information Signals and Endogenous Preferences for Labeled Attributes. J Agric Resour Econ. 2015 May;40(2):179–202.

[23] Lee WJ, Shimizu M, Kniffin KM, Wansink B. You taste what you see: Do organic labels bias taste perceptions? Food Qual Prefer. 2013 7 1;29(1):33–9.

[24] Hughner RS, McDonagh P, Prothero A, Shultz CJ, Stanton J. Who are organic food consumers? A compilation and review of why people purchase organic food. J Consum Behav. 2007 3 1;6(2–3):94–110.

[25] Larceneux F, Benoit-Moreau F, Renaudin V. Why Might Organic Labels Fail to Influence Consumer Choices? Marginal Labelling and Brand Equity Effects. J Consum Policy. 2012 3 1;35(1):85–104.

[26] Vega-Zamora M, Torres-Ruiz FJ, Murgado-Armenteros EM, Parras-Rosa M. Organic as a Heuristic Cue: What Spanish Consumers Mean by Organic Foods. Psychol Mark. 2014 5;31(5):349–59.

[27] Schuldt JP, Muller D, Schwarz N. The “Fair Trade” Effect: Health Halos From Social Ethics Claims. Soc Psychol Personal Sci. 2012 9 1;3(5):581–9.

[28] Lusk JL, Schroeder TC, Tonsor GT. Distinguishing beliefs from preferences in food choice. Eur Rev Agric Econ. 2014 9 1;41(4):627–55.

[29] McFadden B. ‘Gluten-free water’ shows absurdity of trend in labeling what’s absent. The Conversation [Internet]. 2017 Aug 28 [cited 2018 Jul 8]; Available from: http://theconversation.com/gluten-free-water-shows-absurdity-of-trend-in-labeling-whats-absent-80657

[30] Rozin P. Technological Stigma: Some Perspectives from the Study of Contagion. In: Risk, Media and Stigma: Understanding Public Challenges to Modern Science and Technology. 1st Edition. Earthscan; 2001.

[31] Kanter C, Messer KD, Kaiser HM. Does Production Labeling Stigmatize Conventional Milk? Am J Agric Econ. 2009 11 1;91(4):1097–109.

[32] The Non-GMO Project. Product Verification for the Non-GMO Project [Internet]. 2019. Available from: https://www.nongmoproject.org/product-verification/

[33] United States Department of Agriculture (USDA). Organic Certification and Accreditation [Internet]. 2019. Available from: https://www.ams.usda.gov/services/organic-certification

[34] McFadden BR, Malone T How will mandatory labeling of genetically modified food nudge consumer decision-making? J Behav Exp Econ. 2018 12 1;77:186–94.

[35] Lusk JL, Rozan A. Public Policy and Endogenous Beliefs: The Case of Genetically Modified Food. J Agric Resour Econ. 2008;33(2):270–89.

[36] Kolodinsky J, Lusk JL. Mandatory labels can improve attitudes toward genetically engineered food. Sci Adv. 2018 6 1;4(6):eaaq1413 10.1126/sciadv.aaq1413 29963622 PMC6021136

[37] Liaukonyte J, Streletskaya NA, Kaiser HM, Rickard BJ. Consumer Response to “Contains” and “Free of” Labeling: Evidence from Lab Experiments. Appl Econ Perspect Policy. 2013 9 1;35(3):476–507.

[38] Carlsson F, Frykblom P, Lagerkvist CJ Using cheap talk as a test of validity in choice experiments. Econ Lett. 2005 11 1;89(2):147–52.

[39] Loomis J. What’s to Know About Hypothetical Bias in Stated Preference Valuation Studies? J Econ Surv. 2011 4 1;25(2):363–70.

[40] de-Magistris T, Gracia A, Nayga RM. On the Use of Honesty Priming Tasks to Mitigate Hypothetical Bias in Choice Experiments. Am J Agric Econ. 2013 10 1;95(5):1136–54.

[41] Train KE. Discrete Choice Methods with Simulation. Cambridge University Press; 2009. 399 p.

[42] Kahneman D, Tversky A. Prospect Theory: An Analysis of Decision under Risk. Econometrica. 1979;47(2):263–91.

[43] Carlsson F, Mørkbak MR, Olsen SB. The first time is the hardest: A test of ordering effects in choice experiments. J Choice Model. 2012 1 1;5(2):19–37.

